# Reference-signature projection framework for exercise transcriptomic directionality based on the IAV-DI reference axis

**DOI:** 10.3389/fphys.2026.1943015

**Published:** 2026-10-07

**Authors:** Anli Zhou, Min Zhang, Cheng Zhang, Jianjun Guo, Min Zhang, Yan Zhang

**Affiliations:** 1 Center for Innovation in Sports-Medicine Integration, Capital University of Physical Education and Sports, Beijing, China; 2 Beijing Key Laboratory of Interdisciplinary Intelligent Technology of Sports, Medicine and Engineering, Beijing, China; 3 Department of Nephrology, Beijing Chaoyang Hospital, Capital Medical University, Beijing, China

**Keywords:** directional concordance, exercise transcriptomics, gene set enrichment analysis, IAV-DI reference axis, random forest mean decrease in accuracy, reference-signature projection framework

## Abstract

**Background:**

Exercise is often described as medicine, but exercise and pharmacological interventions cannot be treated as clinically equivalent. This study developed a Reference Signature Projection Framework (RSPF) to evaluate the directional relationship of external disease and exercise transcriptomic responses relative to a predefined disease-treatment reference axis. The empirical analysis in this study was restricted to the IAV-DI reference axis defined by dimethyl itaconate (DI) treatment in the context of influenza A virus (IAV) infection.

**Methods:**

(1) Reference-signature construction: the IAV-DI reference direction was defined from transcriptional changes between DI-treated and untreated human peripheral blood mononuclear cells in the context of IAV infection. Drug-UP and Drug-DOWN directions were defined according to avg_log2FC, candidate genes were prioritized using random forest Mean Decrease Accuracy (MDA), and the MDA Top 100 was used as the main reference signature. (2) Reference-signature projection: Drug-UP and Drug-DOWN were projected separately onto complete gene-ranking lists for GSE243629 IA versus HC, cardiopulmonary exercise testing (CPX), and Marathon post- versus pre-exercise comparisons, and GSEA was used to calculate NES, nominal p, and FDR. (3) Robustness and supporting evaluation: gene-level similarity, TopN sensitivity, random gene-set negative controls, and pathway-level NES patterns were used to evaluate transcriptomic directionality, robustness, and interpretive boundaries.

**Results:**

(1) Reference-signature composition: the MDA Top 100 contained 24 Drug-UP and 76 Drug-DOWN genes. (2) External disease-direction evaluation: in GSE243629 IA versus HC, Drug-DOWN was significantly enriched toward the upregulated end (NES = 1.724, FDR = 4.00 × 10^−8^), whereas Drug-UP did not reach FDR< 0.05, indicating a clear opposite relationship of the external disease state relative to the IAV-DI reference axis, with statistical support driven mainly by the Drug-DOWN reference gene set. (3) Overall exercise-direction results: neither Drug-UP nor Drug-DOWN reached FDR< 0.05 in CPX or Marathon; therefore, their direction scores were interpreted only as exploratory directional indicators. Gene-level analysis likewise showed a weak overall directional relationship between CPX and IAV-DI. (4) Pathway-level results: selected functional modules showed the same NES sign for IAV-DI and CPX and the opposite sign for Marathon, but none of the CPX pathways reached FDR < 0.05; these results therefore represent only module-specific exploratory directional patterns.

**Conclusion:**

RSPF provides a computational framework for evaluating transcriptomic directional relationships relative to a predefined disease-treatment reference axis. In the current IAV-DI proof-of-concept analysis, the external influenza disease comparison showed a clear opposite relationship relative to the IAV-DI reference axis, whereas neither CPX nor Marathon showed statistically supported overall IAV-DI directional concordance; exploratory directional patterns were observed only in selected functional modules. Accordingly, the present findings apply only to the IAV-DI reference axis and cannot be generalized to a universal relationship between exercise and pharmacological interventions or used to infer therapeutic equivalence, drug substitution, or shared pharmacological mechanisms.

## 1 Introduction

The concept of “exercise is medicine” highlights the value of physical activity in health promotion and disease prevention. Regular exercise has been associated with lower chronic-disease risk, maintenance of immune homeostasis, and improved metabolic health (Pedersen and Saltin, 2015; Booth et al., 2012; Gleeson et al., 2011; Pedersen and Febbraio, 2012; Catoire and Kersten, 2015). Recent studies further indicate that exercise-induced molecular responses are strongly dependent on time, tissue, and cell type. Peripheral leukocyte metabolism can be modulated by acute and chronic exercise, while multi-tissue multi-omic studies and human PBMC single-cell studies have shown broad and dynamic immune, metabolic, and transcriptional responses to endurance exercise (Hodgman et al., 2023; MoTrPAC Study Group et al., 2024; Yu et al., 2023; Veschetti et al., 2025). Exercise is therefore a systemic stimulus shaped by modality, intensity, duration, training status, and individual variation and should not be treated as a simple equivalent of a drug effect.

To restrict the question to what transcriptomic data can reasonably address, this study focuses on the directional relationship of disease- or exercise-induced gene-expression changes relative to a predefined disease-treatment reference axis, rather than testing whether exercise has the same clinical efficacy as a treatment or inferring shared pharmacological targets. Throughout this manuscript, terms such as “co-directional, “ “opposite-direction, “ or “closer to the reference direction” refer only to transcriptomic directional relationships at the level of whole-gene rankings, GSEA, and gene effect sizes; they do not imply clinical therapeutic equivalence, shared pharmacological mechanisms, or drug substitution.

To address this question, we developed a Reference Signature Projection Framework (RSPF). The framework first constructs a directional reference signature from a predefined disease-treatment comparison and then projects Drug-UP and Drug-DOWN gene sets separately onto complete gene-ranking lists from external disease and exercise comparisons, with interpretation based on GSEA normalized enrichment scores (NES) and gene-level similarity. This strategy is conceptually related to the Connectivity Map approach, which compares perturbations using directional expression signatures (Lamb et al., 2006; Subramanian et al., 2017), but here the purpose is limited to evaluating transcriptomic directionality rather than inferring disease reversal, therapeutic equivalence, or drug substitution.

The transcriptional difference between DI-treated and untreated conditions in the context of IAV infection was selected as a proof-of-concept reference axis. The primary rationale was that GSE164922 provides a direct DI-treated versus untreated comparison within the same IAV-infected context, while the independent dataset GSE243629 provides an IA versus HC disease comparison that can be used to evaluate the direction of an external disease response relative to the same axis. Selection of the IAV-DI axis does not imply that DI is superior to other treatments or that IAV represents exercise physiology in general. The specific objectives were to (1) construct and evaluate the MDA Top 100 reference signature, (2) compare the directional relationships of external disease, CPX, and Marathon responses relative to the IAV-DI reference axis, and (3) localize differences among these transcriptional comparisons at the functional-module level. For other disease-treatment combinations, the corresponding reference signature would need to be reconstructed and independently validated.

## 2 Materials and methods

### 2.1 Study design and data sources

The overall analysis included three categories of publicly available transcriptomic data for IAV-DI reference-signature construction, exercise transcriptomic-response analysis, and external disease-direction evaluation.

#### 2.1.1 IAV-DI reference data

GSE164922 contains 10x Genomics single-cell RNA-seq data from human peripheral blood mononuclear cells (PBMCs), including uninfected control, IAV-infected untreated, IAV-infected DI-treated, and uninfected DI-treated conditions (Sohail et al., 2022). The IAV-infected DI-treated versus IAV-infected untreated comparison was used to define the IAV-DI reference axis.

#### 2.1.2 Exercise data

CPX and Marathon data were obtained from the PBMC single-cell study of Yu et al. (2023). CPX used the Bruce treadmill protocol as a symptom-limited incremental cardiopulmonary exercise test lasting approximately 10–15 min, with workload progressively increased until exhaustion or the relevant termination criteria were reached; high-load indicators reported in the original study included RER > 1.1 or heart rate > 85% of predicted maximal heart rate. Marathon represented prolonged high-load endurance running lasting approximately 4 h. CPX participants had no regular exercise habits, whereas Marathon participants had regular marathon-training backgrounds. The main analysis used Time1 (5 min after exercise) versus Time0 (pre-exercise) for both exercise conditions.

#### 2.1.3 External disease data

GSE243629 was used to evaluate the directional relationship of an external disease transcriptomic response relative to the IAV-DI reference axis. The main comparison was adults with influenza A infection (IA) versus healthy controls (HC) (Zhang et al., 2023).

Data sources, participant information, sampling design, and analytical use are summarized in **Supplementary Table S1**.

### 2.2 RSPF reference-signature construction and transcriptomic projection

RSPF defines the IAV-DI reference axis using IAV_DI_Treated versus IAV_Untreated, constructs Drug-UP and Drug-DOWN reference signatures, and projects these signatures onto external disease and exercise transcriptomic responses (**Figure 1**).

**Figure 1:**
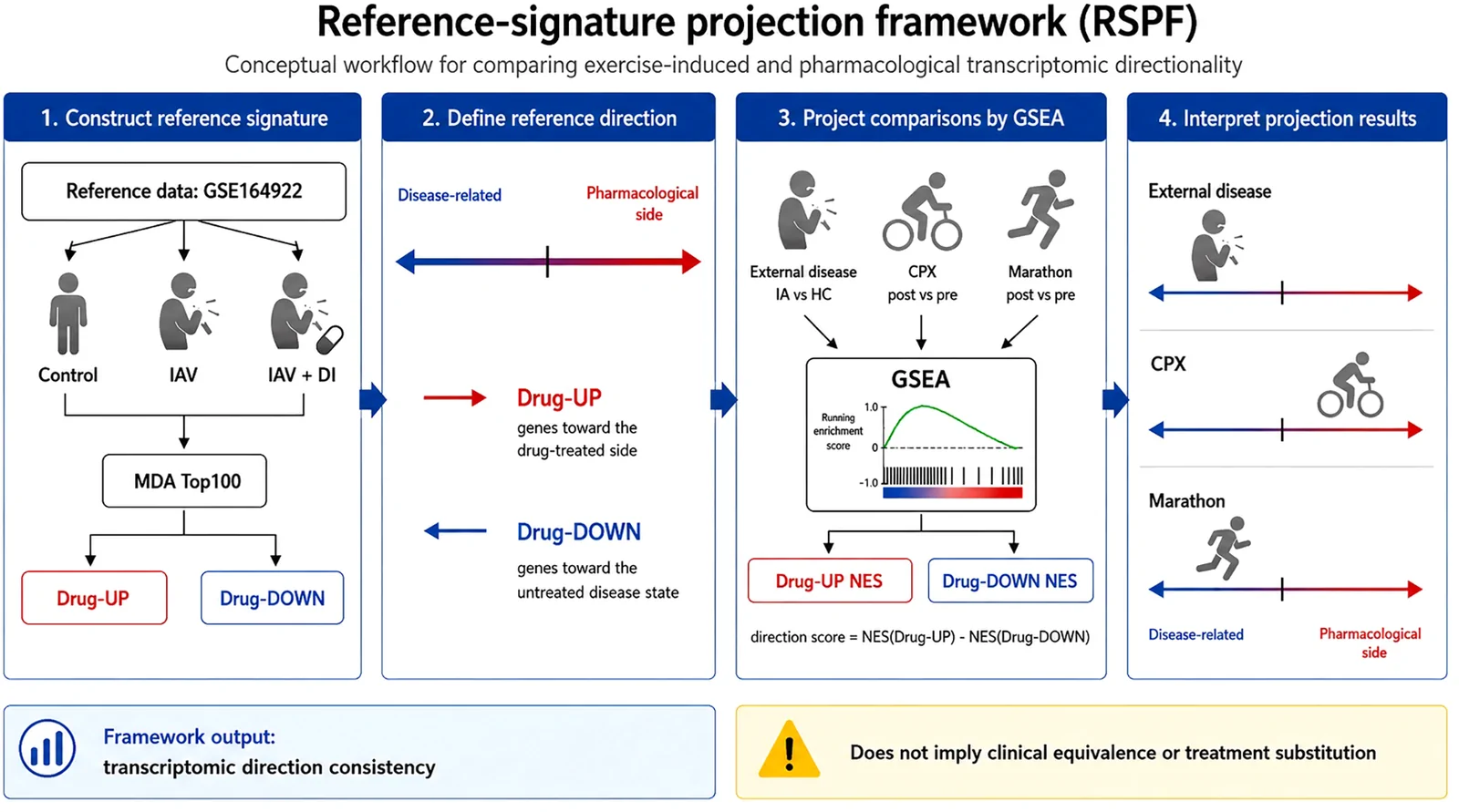
Overall principle of the reference signature projection framework. The figure summarizes construction of the IAV-DI reference signature, definition of Drug-UP and Drug-DOWN, GSEA-based projection onto external disease and exercise comparisons, and conservative interpretation using direction score and gene-level similarity. Framework outputs represent transcriptomic directional relationships only and do not imply clinical equivalence or treatment substitution.

1. Reference-gene screening and direction definition. Differential expression analysis was performed using the default Wilcoxon rank-sum test in Seurat FindMarkers (logfc.threshold = 0.15, min.pct = 0.1; Hao et al., 2021), and the top 1, 000 candidates from the differential-expression ranking were entered into random-forest analysis. Genes with avg_log2FC > 0 were defined as Drug-UP, whereas genes with avg_log2FC< 0 were defined as Drug-DOWN. Because IAV_DI_Treated and IAV_Untreated each corresponded to only one original 10x sample, the cell-level analysis was used only for candidate screening and direction definition and not for donor-level statistical inference. This treatment is consistent with recent methodological work emphasizing biological replication and pseudoreplication in single-cell differential-expression analysis (Zimmerman et al., 2021; Squair et al., 2021; Junttila et al., 2022; Murphy and Skene, 2022). A separate count-aggregation analysis was used to assess the stability of Drug-UP/Drug-DOWN direction under an alternative expression summarization strategy; details are provided in **Supplementary Table S13**.
2. MDA ranking and reference-signature definition. A random-forest classifier was fitted with the randomForest package (ntree = 500, seed = 42, importance = TRUE; default classification mtry = sqrt(p); Breiman, 2001; Liaw and Wiener, 2002). Mean Decrease Accuracy (MDA) was used to prioritize candidate genes (Strobl et al., 2007; Altmann et al., 2010), and the MDA Top 100 was selected as the main reference signature. MDA determined only the priority ranking of candidate genes; Drug-UP and Drug-DOWN direction remained defined by avg_log2FC. The DEG-only comparison used the same differential-expression results and direction rules but omitted MDA re-ranking and served as a methodological control (**Figure 2**).
3. Whole-gene ranking and GSEA projection. CPX, Marathon, and GSE243629 were each represented by complete gene-ranking lists constructed from processed expression data. The ranking metric for each gene was defined as:

**Figure 2:**
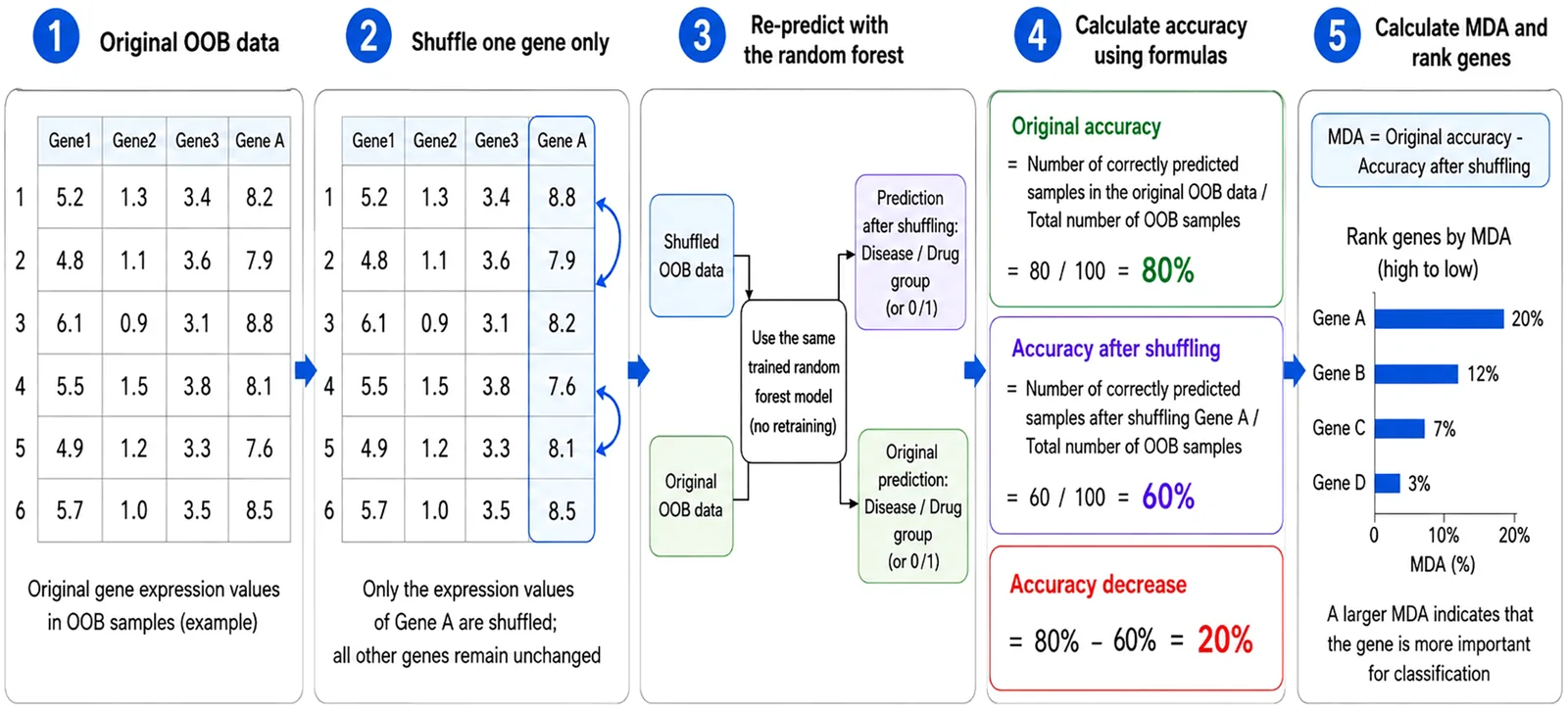
Schematic workflow of random forest mean decrease accuracy (MDA). MDA is used only to rank candidate genes by permutation importance; it does not determine gene direction and does not prespecify the Drug-UP/Drug-DOWN ratio.

Specifically, CPX and Marathon used sample_suffix and GSE243629 used sample_id as the analytical aggregation identifier. Mean normalized expression from the Seurat RNA assay was calculated within each aggregation unit, with seed = 42 and a maximum of 10, 000 cells retained per comparison condition.


$$rank\_score_{g}=\bar{E}\left(g,A\right)-\bar{E}\left(g,B\right)$$

For CPX and Marathon, the comparison was Time1 versus Time0; for GSE243629, the comparison was IA versus HC. All genes with valid rank_score values were ordered from highest to lowest without pre-filtering by single-gene p values or FDR. Drug-UP and Drug-DOWN were then analyzed separately using preranked GSEA, and NES, nominal p, and FDR were recorded (Subramanian et al., 2005; Korotkevich et al., 2016). The direction score was defined as:

A complete gene ranking was used because preranked GSEA evaluates gene-set enrichment toward either end of a continuous genome-wide ranking (Subramanian et al., 2005); pre-filtering by single-gene p values or FDR may discard gene-set signals with modest effect sizes but coordinated directionality. Cell-level Wilcoxon statistics were also not used directly as the target ranking metric, to avoid introducing significance structures related to cell number and pseudoreplication into cross-dataset directional comparisons (Zimmerman et al., 2021; Squair et al., 2021). Therefore, the condition mean normalized-expression difference was used to construct a continuous ranking that primarily reflects direction and relative effect magnitude.


Direction score=NES(Drug-UP)−NES(Drug-DOWN)

The direction score is used only to describe the relative enrichment pattern of the Drug-UP and Drug-DOWN reference gene sets and is interpreted together with the separate NES, nominal p, and FDR values for Drug-UP and Drug-DOWN.

### 2.3 Supporting analyses

To evaluate the robustness and interpretive boundaries of the main results, the following supporting analyses were performed:

1. Gene-level similarity: effect values of the MDA Top 100 genes were compared between the IAV-DI reference comparison and each target comparison, and Spearman correlation, Pearson correlation, and cosine similarity were calculated (**Figure 3**).
2. TopN sensitivity: GSEA was repeated using MDA Top 50, Top 100, and Top 200 signatures to assess sensitivity to the number of reference genes.
3. Random gene-set negative controls: 1, 000 random gene sets of matched size were sampled and the observed NES values were compared with the random background.
4. MDA/DEG-only comparison: gene composition, overlap, and functional-enrichment characteristics were compared between the two ranking strategies to evaluate differences introduced by reference-signature construction.

**Figure 3:**
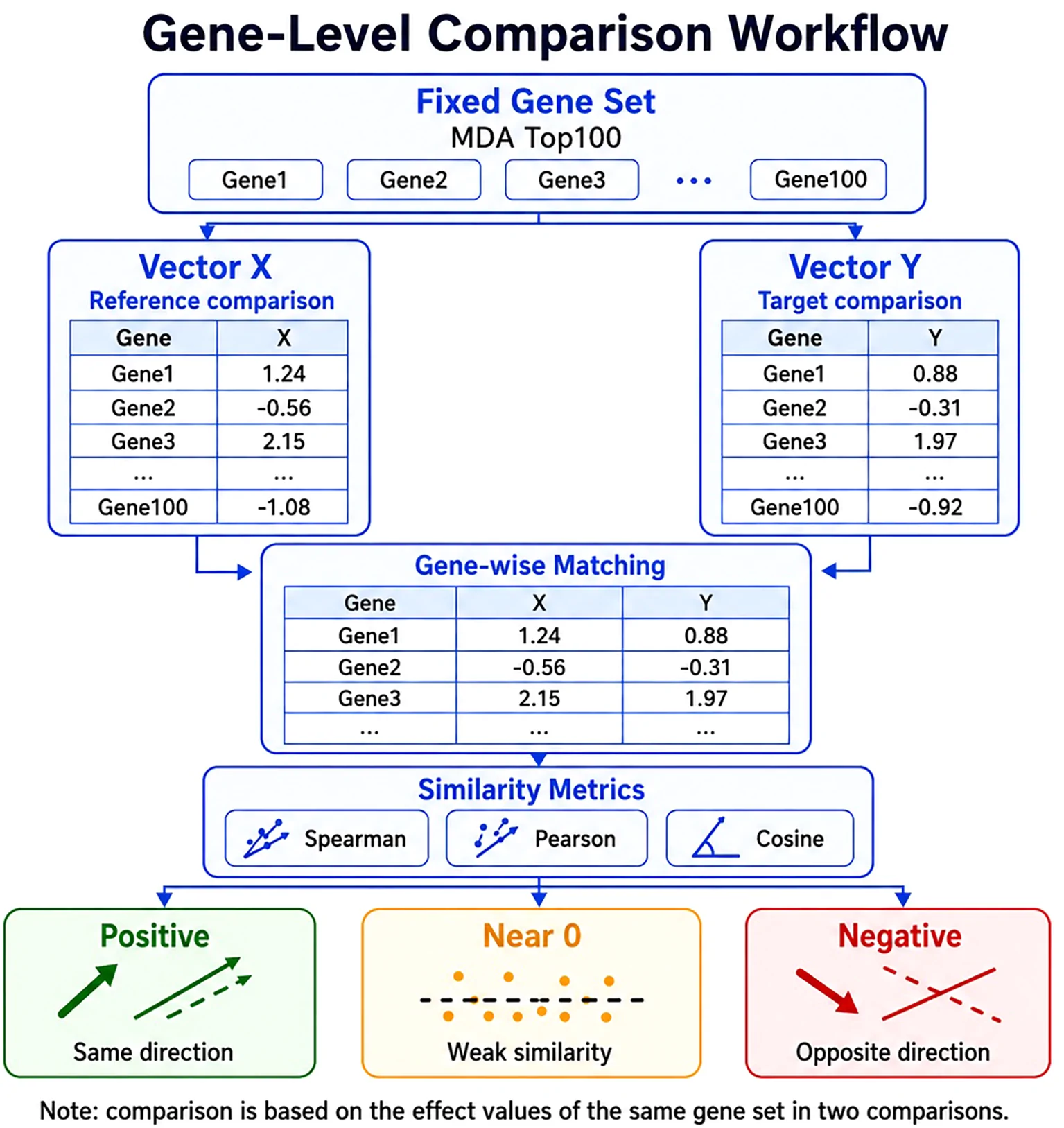
Principle of gene-level similarity analysis. Effect sizes of the MDA Top 100 genes are paired between the IAV-DI reference comparison and each external disease/exercise comparison, and Spearman correlation, Pearson correlation, and cosine similarity are calculated.

Detailed parameters are provided in **Supplementary Table S2**.

### 2.4 Pathway-level enrichment-direction comparison

On the basis of the global RSPF projection and gene-level similarity analyses, Hallmark and Reactome GSEA was further performed using the complete gene rankings for IAV-DI, CPX, and Marathon to compare enrichment directions in specific pathways (Liberzon et al., 2015; Milacic et al., 2024). NES was used to describe enrichment direction and FDR to evaluate statistical support. This analysis was used to identify local co-directional or opposite-direction relationships within specific pathways and was not treated as independent evidence of global transcriptomic concordance.

## 3 Results

### 3.1 Directional composition of the MDA top 100 reference signature

The MDA Top 100 contained 24 Drug-UP and 76 Drug-DOWN genes; by contrast, the DEG-only Top 100 contained 4 Drug-UP and 96 Drug-DOWN genes, and the DEG-only Top 50 contained only 1 Drug-UP gene. This difference resulted from MDA re-ranking candidates according to multivariate discriminatory contribution rather than from any predefined adjustment of the Drug-UP/Drug-DOWN ratio. Some Drug-UP genes that ranked lower in single-gene differential-expression results had higher discriminatory contribution in the multivariate model separating IAV_DI_Treated from IAV_Untreated and therefore received higher priority under MDA. Because RSPF projects Drug-UP and Drug-DOWN as two separate gene sets, an extremely small gene set in one direction is more sensitive to a few individual genes and is less suitable for stable two-set comparison. The 24/76 composition therefore arose naturally from MDA re-ranking; no Drug-UP/Drug-DOWN proportion was prespecified (**Figure 4**).

**Figure 4:**
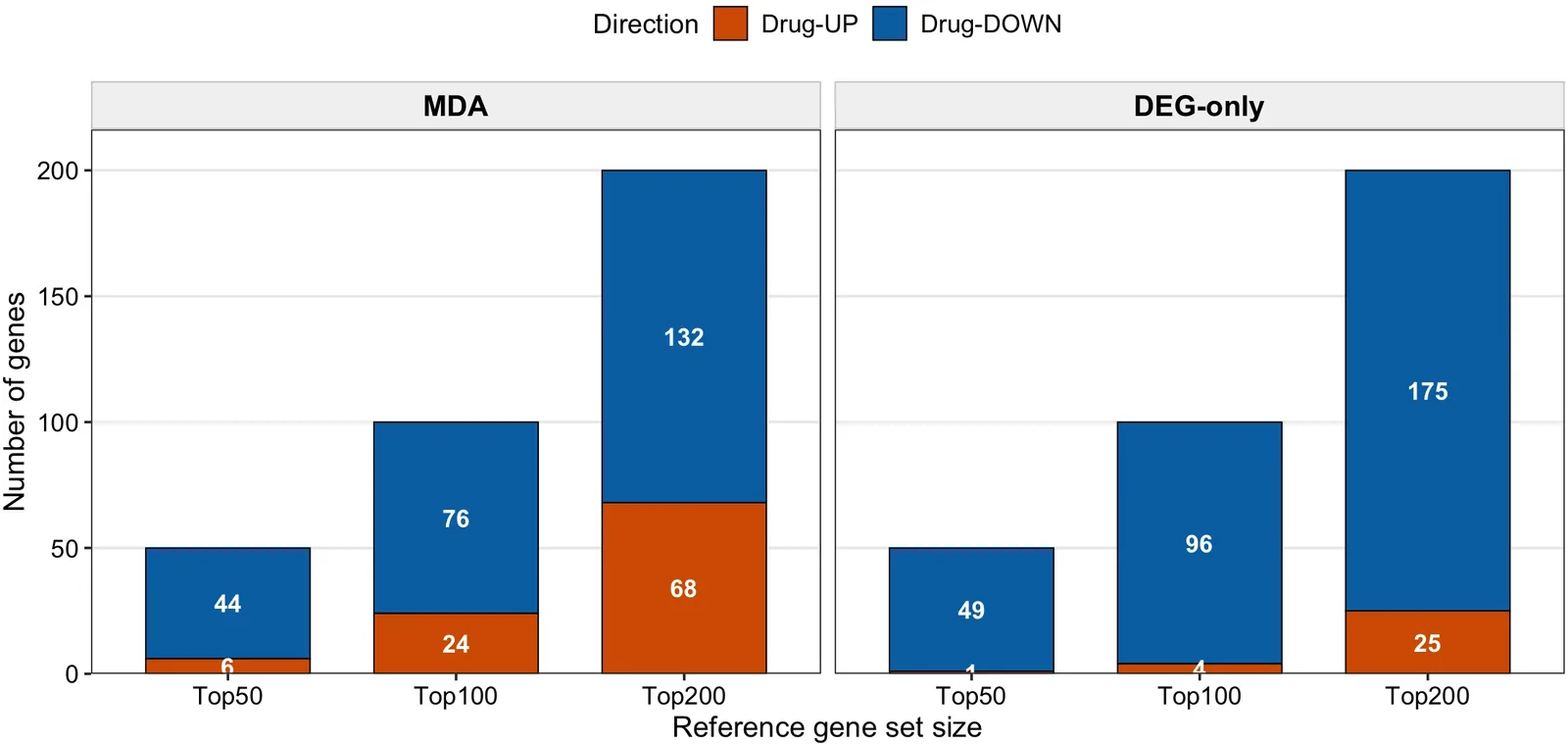
Directional composition of MDA and DEG-only reference signatures. DEG-only Top 50 and Top 100 are strongly dominated by Drug-DOWN, whereas MDA Top 100 contains 24 Drug-UP and 76 Drug-DOWN genes. This composition is an observed result of MDA ranking rather than a prespecified balancing ratio.

IAV_DI_Treated and IAV_Untreated each corresponded to one original 10x sample and therefore lacked independent biological replication for conventional donor-level pseudobulk inference. To assess whether Drug-UP/Drug-DOWN direction depended on the cell-level expression representation, counts from each condition were separately aggregated and gene-change directions were recalculated and compared with cell-level avg_log2FC directions. Of 3, 236 comparable reference genes, 3, 223 retained the same direction, corresponding to 99.60% concordance; Spearman and Pearson correlations between the two effect representations were 0.951 and 0.983, respectively. These results support high within-dataset stability of Drug-UP/Drug-DOWN direction to expression summarization. Full results, including the 13 genes whose directions changed, are provided in **Supplementary Table S13**.

### 3.2 External disease-direction evaluation and exercise-direction comparison

Within the RSPF projection, a target comparison aligned with the IAV-DI reference direction would be expected to show Drug-UP enrichment toward the upregulated end (NES > 0) and Drug-DOWN enrichment toward the downregulated end (NES< 0). The opposite NES combination indicates an opposite relationship to the IAV-DI reference direction. Statistical support for each enrichment direction was evaluated using its nominal p value and FDR.

For GSE243629 IA versus HC, Drug-UP NES was -1.278 (nominal p = 0.092, FDR = 0.092) and Drug-DOWN NES was 1.724 (nominal p = 2.00 × 10^−8^, FDR = 4.00 × 10^−8^), yielding a direction score of -3.002. Drug-UP was enriched toward the downregulated end and Drug-DOWN toward the upregulated end, giving an overall NES combination opposite to the IAV-DI reference direction. However, statistical support was driven primarily by the significant Drug-DOWN enrichment, whereas Drug-UP did not reach FDR< 0.05. Thus, the result supports an overall negative directional relationship of the external disease comparison relative to the IAV-DI reference axis rather than a fully significant bidirectional reversal. Main GSEA results are shown in **Figure 5** and **Supplementary Table S4**.

**Figure 5:**
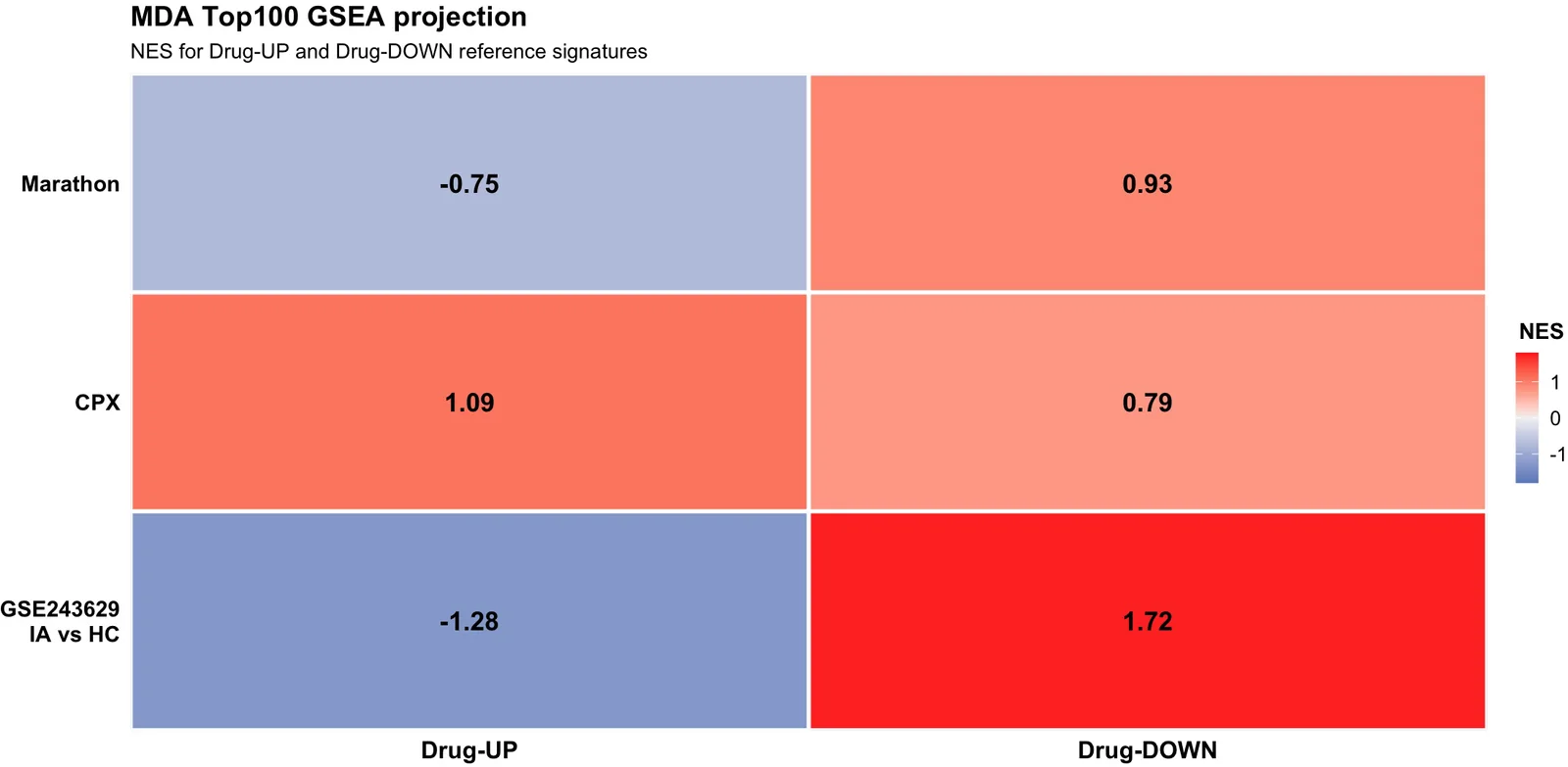
GSEA NES heatmap of the MDA Top 100 reference signature. Color represents NES direction and magnitude; nominal p values and FDR values for each Drug-UP and Drug-DOWN enrichment are reported in the main text and **Supplementary Table S4**.

For CPX, Drug-UP NES was 1.094 (nominal p = 0.356, FDR = 0.713) and Drug-DOWN NES was 0.790 (nominal p = 0.842, FDR = 0.842), yielding a direction score of 0.304. Although Drug-UP had the same enrichment direction expected under the IAV-DI reference axis, Drug-DOWN was also enriched toward the upregulated end, so the coordinated Drug-UP-positive/Drug-DOWN-negative pattern was not observed. Neither gene set reached FDR< 0.05. CPX therefore did not show statistically supported overall IAV-DI directional concordance.

For Marathon, Drug-UP NES was -0.747 (nominal p = 0.812, FDR = 0.812) and Drug-DOWN NES was 0.925 (nominal p = 0.599, FDR = 0.812), yielding a direction score of -1.672. The NES combination was opposite to the IAV-DI reference direction, but neither gene set reached FDR< 0.05; accordingly, this result was interpreted only as an exploratory opposite-direction pattern without clear statistical support. **Figure 6** provides a descriptive visualization of direction scores across the three comparisons; statistical interpretation remains based on the separate GSEA results for Drug-UP and Drug-DOWN.

**Figure 6:**
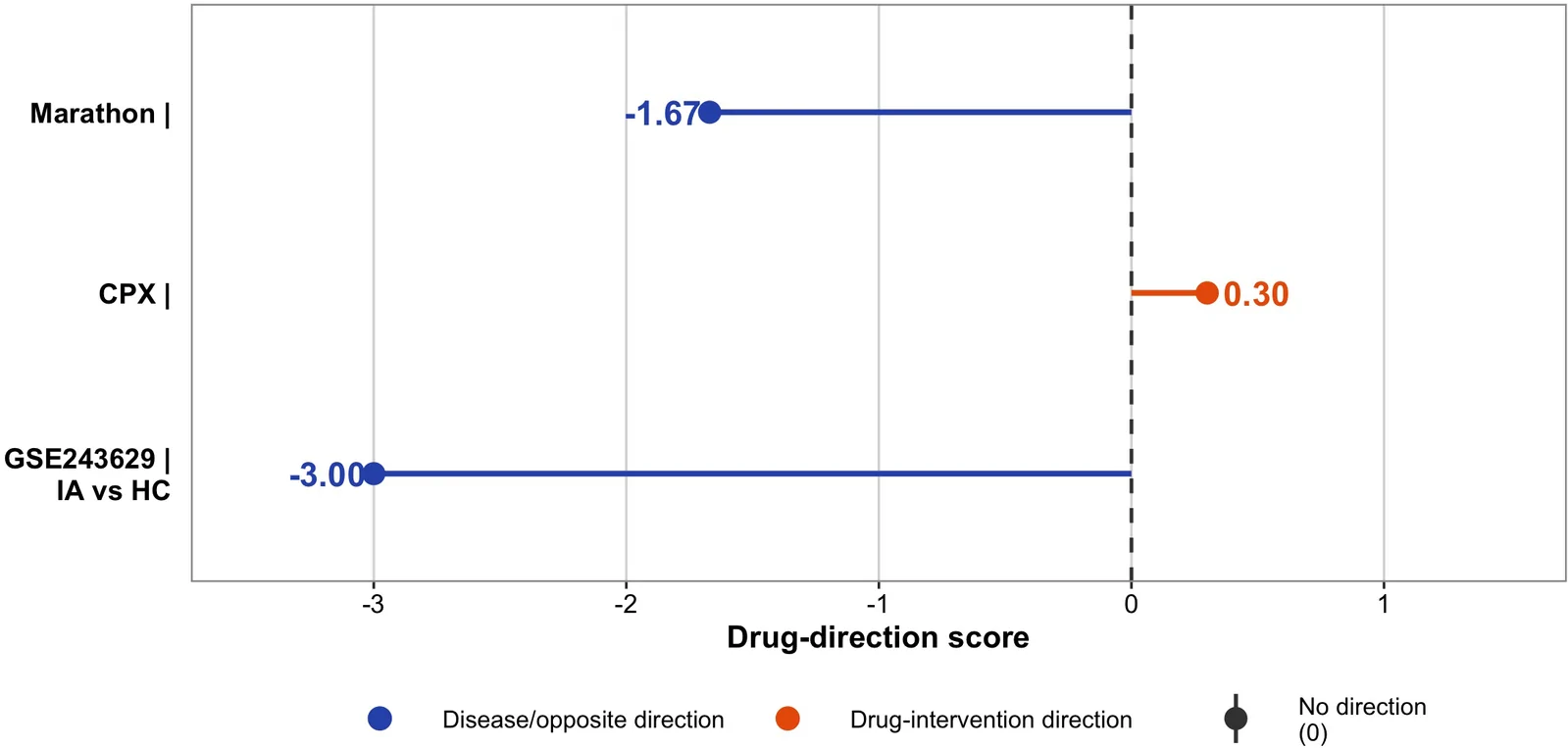
Descriptive direction scores relative to the IAV-DI reference axis. Direction score = NES(Drug-UP) − NES(Drug-DOWN) and is used only to summarize the relative pattern of the two reference gene sets. It is not an independent statistical test; statistical support is determined from the separate GSEA nominal p and FDR values for Drug-UP and Drug-DOWN.

### 3.3 Gene-level similarity and robustness analyses

Gene-level similarity analysis showed that the Spearman correlation, Pearson correlation, and cosine similarity between GSE243629 IA versus HC and the IAV-DI reference comparison were -0.57, -0.56, and -0.73, respectively, indicating an overall negative association in gene effect sizes. The corresponding values for CPX were 0.05, 0.13, and 0.03, indicating a weak overall directional relationship with the IAV-DI reference comparison. For Marathon, the corresponding values were -0.10, -0.13, and -0.16, likewise indicating a weak overall directional relationship without a clear concordant or opposite pattern (**Table 1**; **Figure 7**).

**Table 1:** Gene-level similarity of MDA Top 100 reference genes between the IAV-DI reference comparison and external disease/exercise comparisons.

Comparison	Spearman correlation	Pearson correlation	Cosine similarity	Interpretation
GSE243629:IA vs HC	−0.57	−0.56	−0.73	Overall negative association in gene effect sizes
CPX	0.05	0.13	0.03	Weak overall directional relationship with the IAV-DI reference comparison.
Marathon	−0.10	−0.13	−0.16	Weak overall directional relationship with the IAV-DI reference comparison.

**Table 2:** Functional interpretation of pathway modules showing directional differences between CPX and marathon.

Functional module	Representative pathways	Core biological function	Interpretation in this study
TLR/IRAK4/MyD88 innate immune-recognition module	Regulation of TLR by endogenous ligand; IRAK4-related TLR2/4 signaling; Toll-like receptor cascades; TLR1/TLR2; TLR9	Recognition of PAMP/DAMP signals and activation of NF-κB/IRF-related inflammatory and interferon responses (Akira et al., 2006; Takeuchi and Akira, 2010).	IAV-DI and CPX have the same NES sign, whereas Marathon has the opposite sign; CPX did not reach FDR< 0.05 and is therefore interpreted only as an exploratory module-specific directional pattern.
Neutrophil effector/degranulation module	Neutrophil degranulation	Release of granule proteins, proteases, and oxidative-stress-related effector molecules involved in host defense and inflammatory injury (Amulic et al., 2012; Neubauer et al., 2013).	In this module, CPX and IAV-DI have the same sign whereas Marathon has the opposite sign; this difference can localize exercise-mode differences in effector-cell responses but does not indicate that CPX reproduces DI effects.
Antigen-presentation module	MHC class II antigen presentation	Antigen processing and presentation to CD4+ T cells, linking innate and adaptive immunity (Neefjes et al., 2011).	This module shows a local directional difference; CPX enrichment did not reach FDR< 0.05, so statistically supported pathway concordance cannot be inferred.
Integrated immune-system/inflammatory module	Diseases of the Immune System; Reactome Immune System pathways; Hallmark inflammatory and IL6-related pathways	Integrates innate immune recognition, cytokine signaling, effector-cell responses, and interfaces with adaptive immunity.	Used to summarize directional differences among the three independent transcriptomic comparisons in selected immune-related modules; not extrapolated to global transcriptomic or mechanistic concordance.

**Figure 7:**
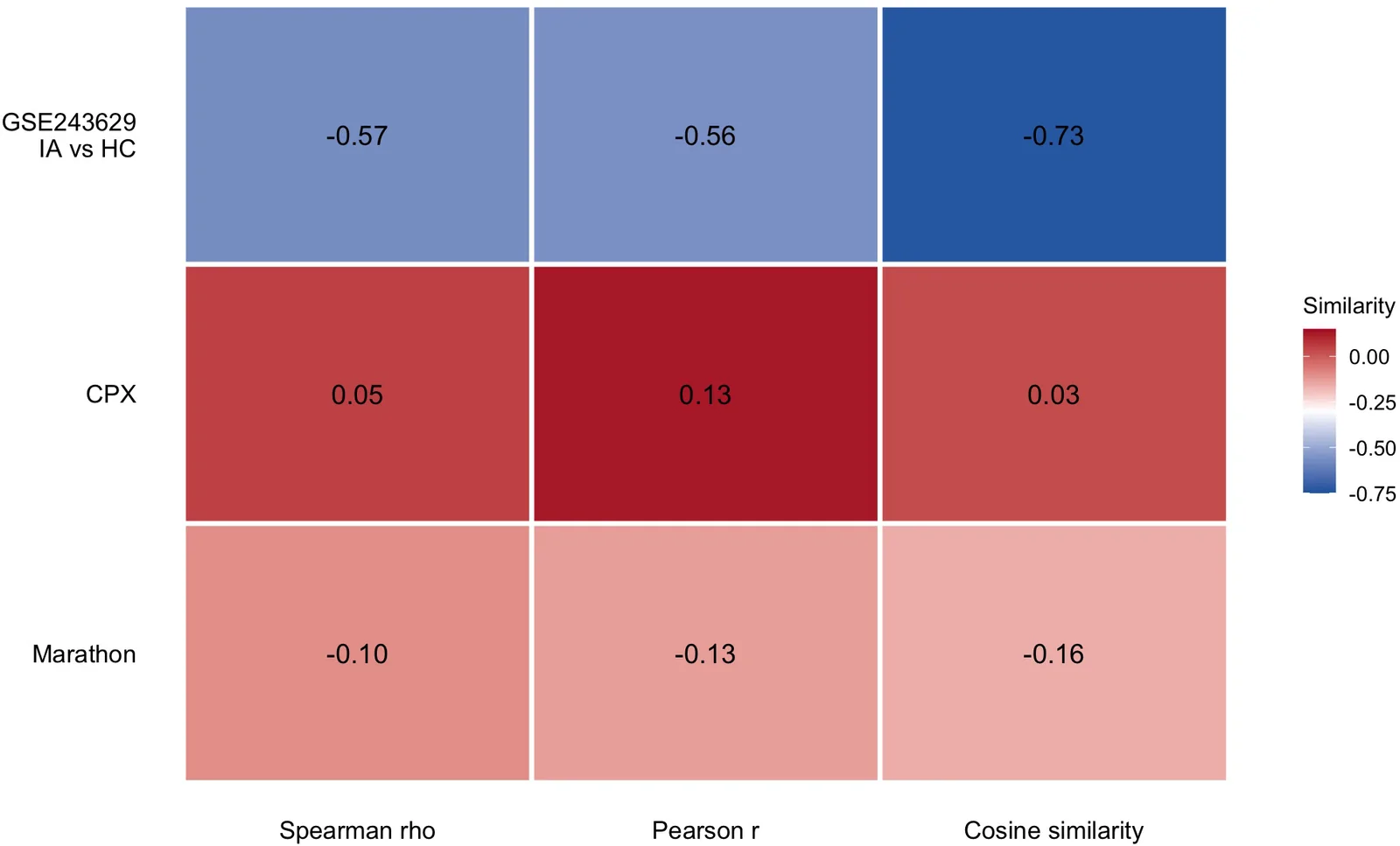
Gene-level similarity of MDA Top 100 reference genes. Spearman correlation, Pearson correlation, and cosine similarity are shown for GSE243629 IA versus HC, CPX, and Marathon relative to the IAV-DI reference comparison.

TopN sensitivity analysis showed CPX direction scores of 0.028, 0.304, and 0.210 for Top 50, Top 100, and Top 200, respectively. Drug-UP and Drug-DOWN NES values were positive at all three TopN settings, so the expected IAV-DI Drug-UP-positive/Drug-DOWN-negative pattern was not reproduced. Marathon direction scores were 0.126, -1.672, and -0.159, and Drug-UP NES changed sign across TopN settings, indicating greater sensitivity to reference-signature size.

In 1, 000 matched-size random gene-set controls, the empirical proportions reaching or exceeding the observed absolute NES were 0.354 and 0.838 for CPX Drug-UP and Drug-DOWN and 0.830 and 0.587 for Marathon Drug-UP and Drug-DOWN, respectively, indicating that the exercise enrichment magnitudes were not clearly separated from the random-gene background. By contrast, for GSE243629 IA versus HC, none of the 1, 000 random controls reached or exceeded the observed absolute Drug-DOWN NES of 1.724 (0/1000; +1-corrected empirical p approximately 0.001), providing additional robustness support for the disease-related Drug-DOWN enrichment. Overall, CPX and Marathon directional findings were treated as exploratory.

### 3.4 Local pathway-level directional patterns and differences between exercise modes

The preceding global RSPF projection and gene-level similarity analyses both indicated a weak overall transcriptomic relationship between CPX and IAV-DI. Hallmark and Reactome GSEA was therefore used to examine whether local functional-module patterns could nevertheless be observed within this globally weak relationship. **Figure 8** displays a representative subset of pathways classified by NES-sign combinations; the counts and proportions describe only the displayed subset and do not represent the overall distribution of all Hallmark and Reactome pathways.

**Figure 8:**
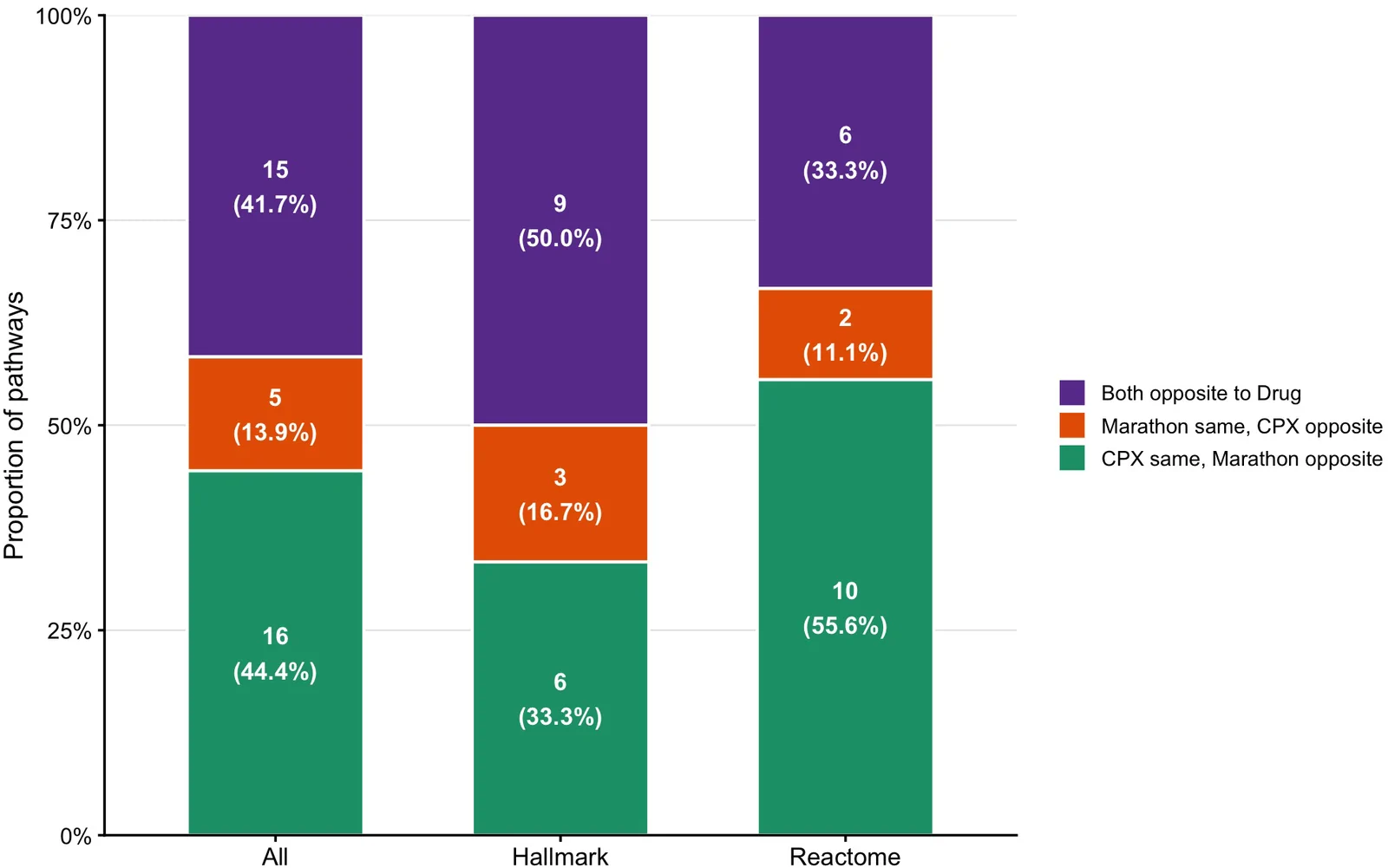
Descriptive display of pathway NES-sign patterns. A representative subset of Hallmark and Reactome pathways is shown and classified by the NES-sign combinations of CPX and Marathon relative to IAV-DI. Counts and proportions within bars refer only to this displayed subset and do not represent the overall distribution of all Hallmark and Reactome pathways.

**Figure 9** further displays 13 representative pathways in which IAV-DI and CPX have the same NES sign, Marathon has the opposite sign, and $\left|NES_{CPX}-NES_{IAV-DI}\right|\leq 1$ is satisfied. These pathways include selected Hallmark processes and Reactome pathways related to TLR signaling, neutrophil degranulation, and MHC II antigen presentation (**Table 2**). IAV-DI reached FDR< 0.05 in 12/13 pathways, Marathon in 2/13, and CPX in 0/13. Therefore, the same-sign NES values for IAV-DI and CPX represent only module-specific exploratory directional patterns and do not constitute statistically supported pathway concordance.

**Figure 9:**
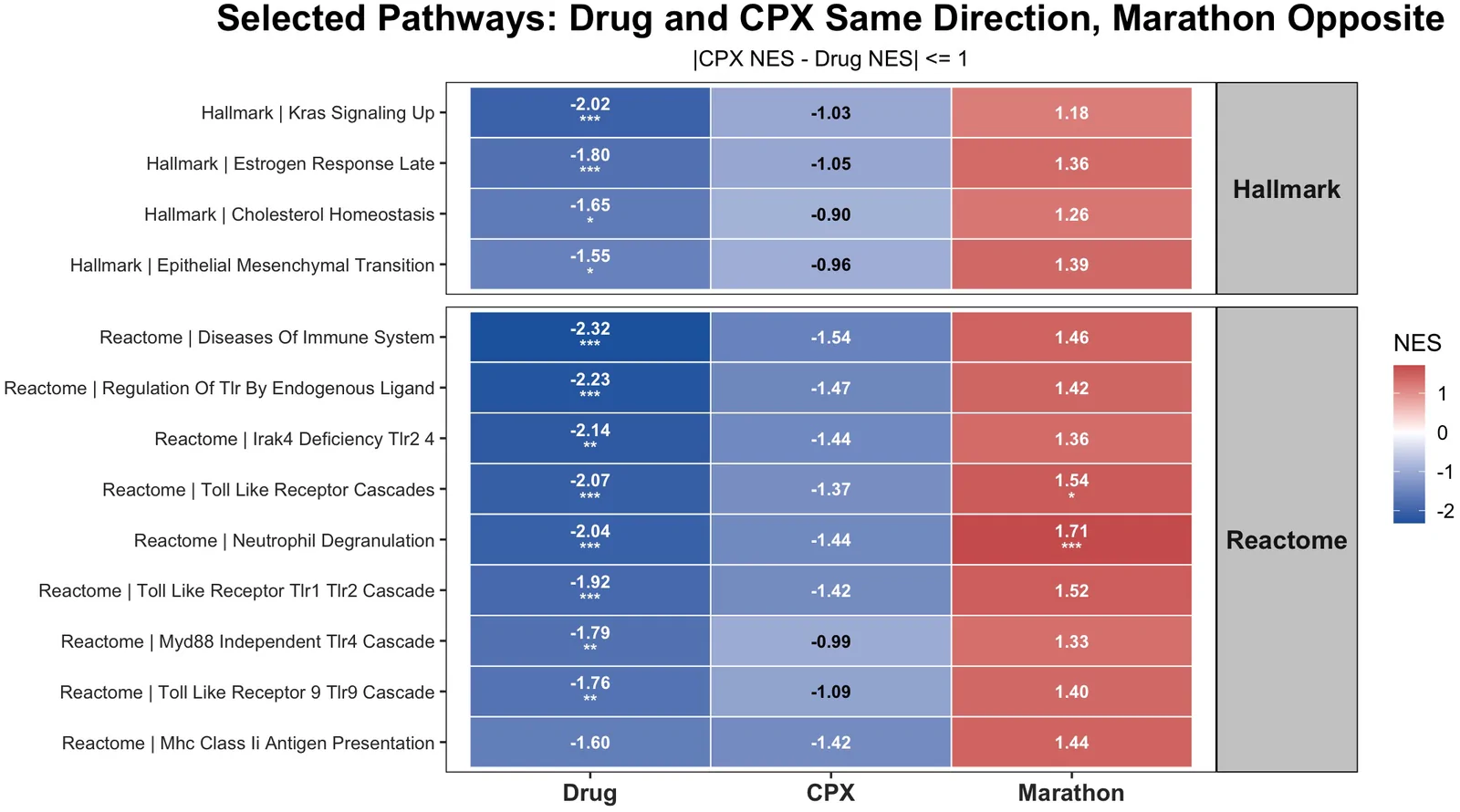
Thirteen representative pathways with the same NES sign for IAV-DI and CPX and the opposite sign for Marathon. Display criteria were sign[NES(IAV-DI)] = sign[NES(CPX)] ≠ sign[NES(Marathon)] and the descriptive range |NES(CPX) − NES(IAV-DI)| ≤ 1. The ≤1 criterion is not a threshold for statistical significance, biological similarity, or equivalence. Asterisks denote FDR-adjusted significance: *FDR< 0.05, **FDR< 0.01, ***FDR< 0.001. None of the 13 displayed CPX pathways reached FDR< 0.05; same-sign CPX and IAV-DI NES values are therefore interpreted only as exploratory module-specific directional patterns.

**Figure 10** further ranks pathways according to $\left|NES_{CPX}-NES_{Marathon}\right|$. Pathways with larger differences mainly involved neutrophil degranulation, TLR-related signaling, MHC II antigen presentation, and immune-system-related processes. This result describes pathway-level differences between CPX and Marathon and is not used to judge which exercise mode is closer to the IAV-DI reference axis.

**Figure 10:**
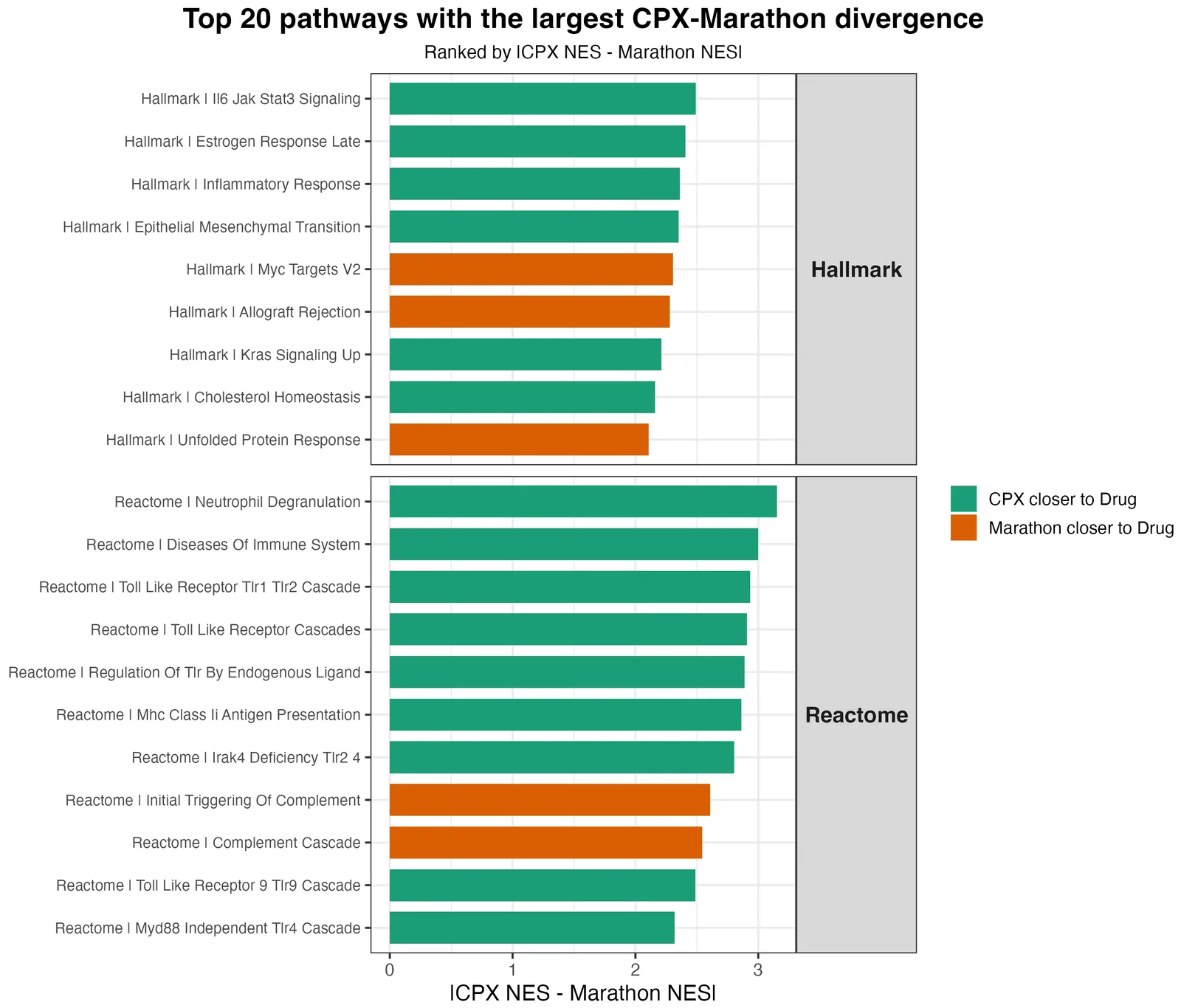
Twenty pathways with the largest CPX-Marathon NES differences. Pathways are ranked by |NES(CPX) − NES(Marathon)| to describe the magnitude of functional-module divergence between the two exercise comparisons; this figure is not used to judge which exercise mode is closer to the IAV-DI reference axis.

## 4 Discussion

### 4.1 Significance of the reference signature projection framework

The main contribution of this study is to transform the broad statement “exercise is medicine” into a restricted transcriptomic question: what directional relationship do external disease or exercise responses show relative to a predefined disease-treatment reference axis? RSPF projects target complete gene rankings into a common framework using Drug-UP and Drug-DOWN reference gene sets and restricts interpretation to the transcriptomic level. The study therefore does not test whether exercise is equivalent to treatment and does not interpret directional similarity as evidence of clinical efficacy or drug substitution.

During reference-signature construction, MDA was used only to prioritize candidate genes. Its permutation importance reflects the decrease in random-forest accuracy for distinguishing untreated IAV infection from DI-treated IAV infection after a given gene is perturbed, but it does not redefine gene direction or create a clinical prediction model. The MDA Top 100 should therefore be understood as a data-driven prioritized reference signature rather than a “biologically optimal” signature. Its composition may depend on the training data, correlation structure among predictors, and model settings, and this selection dependence requires validation in independent data.

Interpretation of RSPF requires separation of multiple evidence levels. The sign of a GSEA NES describes enrichment direction, nominal p and FDR provide statistical support for Drug-UP and Drug-DOWN separately, and the direction score only summarizes the relative pattern of the two NES values. Gene-level similarity evaluates the directional relationship of effect sizes for the same reference genes, while pathway-level analysis localizes signals to specific functional modules. These levels should not be combined into a second, stronger form of “treatment-like” evidence. Functional-enrichment findings should be interpreted together with enrichment direction, multiple-testing correction, and statistical support, and strong biological conclusions should not be drawn from the direction of non-significant pathways alone (Wijesooriya et al., 2022; Chicco and Agapito, 2022). Accordingly, GSEA or pathway results that did not reach FDR significance were treated only as exploratory directional relationships.

### 4.2 Exercise modes and the IAV-DI reference direction

The empirical reference axis in this study was derived from the expression change induced by DI treatment relative to untreated IAV-infected cells. DI is a cell-permeable derivative of itaconate, and previous work indicates that itaconate and its derivatives can modulate IAV-related interferon and inflammatory responses and involve immunometabolic and KEAP1-NRF2-related processes (Sohail et al., 2022; Mills et al., 2018; O'Neill and Artyomov, 2019). The reference direction should therefore be understood as the transcriptomic axis formed by a specific DI intervention in the context of IAV infection, rather than as a general anti-inflammatory drug direction.

At the pathway level, the globally weak CPX-IAV-DI relationship still contained local modules in which IAV-DI and CPX had the same NES sign and Marathon the opposite sign. These modules included TLR-related signaling, neutrophil degranulation, MHC class II antigen presentation, and selected Hallmark processes. Because none of the displayed CPX pathways reached FDR< 0.05, these findings should be treated as module-specific exploratory patterns and cannot be used to infer that CPX reproduces DI-related pathway effects, shares its pharmacological targets, or has therapeutic equivalence.

From an exercise-physiology perspective, CPX is a short-duration progressive-load stimulus, whereas Marathon represents prolonged high-load endurance exercise. The two differ substantially in duration, training background, metabolic stress, immune-cell redistribution, and recovery processes. Recent human PBMC single-cell studies show time-dependent and cell-type-specific transcriptional responses after prolonged endurance exercise (Yu et al., 2023; Veschetti et al., 2025), and exercise can also influence peripheral leukocyte energy metabolism and immune state (Hodgman et al., 2023). The pathway results are therefore more appropriately used to localize directional differences between the two exercise modes in functions such as innate immune recognition, effector-cell responses, and antigen presentation than to establish mechanistic equivalence between exercise and DI. Marathon-associated peripheral blood transcriptional changes may additionally reflect exercise load, tissue damage, metabolic depletion, and recovery-phase immune responses (Neubauer et al., 2013; Hodgman et al., 2023; Yu et al., 2023; Veschetti et al., 2025). Interpretation of CPX-Marathon pathway NES differences should therefore consider exercise-load and sampling-time context and should not extrapolate a single post-exercise time point to long-term adaptation or therapeutic effects.

### 4.3 Limitations and future directions

This study has several limitations.

#### 4.3.1 Scope of the reference axis is limited

The current empirical analysis is based only on the IAV-DI disease-treatment reference axis; the observed directional relationships therefore apply only to this specific reference context and cannot be generalized to a universal transcriptomic relationship between exercise and pharmacological interventions.

#### 4.3.2 Cross-study data are heterogeneous

Although GSE164922, GSE243629, CPX, and Marathon all involve peripheral blood/PBMC-related single-cell transcriptomic data, they differ in study populations, disease context, sequencing and library-preparation systems, sampling time, exercise load, training background, and immune-cell composition. Recent single-cell methodological studies show that batch effects, sequencing depth, sparsity, and integration strategy can influence cross-sample analysis and differential-expression results (Luecken et al., 2022; Nguyen et al., 2023; Heumos et al., 2023). Accordingly, this study compares directional relationships of different transcriptomic responses relative to the same IAV-DI reference axis rather than absolute effect sizes under strictly matched experimental conditions.

#### 4.3.3 The IAV-DI reference data lack independent biological replication

IAV_DI_Treated and IAV_Untreated each corresponded to only one original 10x sample; therefore, cell-level Wilcoxon analysis was used only for candidate-gene screening and direction definition and cannot provide donor-level statistical inference. Cells from the same sample cannot be treated as independent biological replicates, as repeatedly emphasized in recent single-cell differential-expression methodology (Zimmerman et al., 2021; Squair et al., 2021; Junttila et al., 2022; Hafner et al., 2025). The count-aggregation sensitivity analysis can be used to evaluate within-dataset directional stability but cannot compensate for the lack of independent biological replication.

#### 4.3.4 The reference signature is subject to selection dependence

MDA is a data-driven variable-importance ranking method whose results may depend on the training data, candidate-gene set, gene-correlation structure, and model settings. Therefore, the MDA Top 100 should be interpreted as a prioritized reference signature in the current IAV-DI data context rather than as a unique or universally optimal reference gene set.

Future work should reconstruct reference axes in additional disease-treatment combinations with independent biological replication and validate reference-signature stability in independent cohorts. Additional exercise modes and sampling time points, together with cell-composition adjustment and proteomic, metabolomic, or other multi-omic data, could be used to evaluate the cross-cohort and cross-platform reproducibility of local directional patterns.

## Data Availability

Publicly available datasets were analyzed in this study. This data can be found here: https://www.ncbi.nlm.nih.gov/geo/query/acc.cgi?acc=GSE164922,https://www.ncbi.nlm.nih.gov/geo/query/acc.cgi?acc=GSE243629, https://www.biosino.org/node/project/detail/OEP003910.

## Supplementary material

The **Supplementary Material** includes **Supplementary Figures S1**–**S6** and **Supplementary Tables S1**-**S13**. **Supplementary Figures S1**–**S6** comprise TopN sensitivity analysis, gene-level similarity, random gene-set negative controls, overlap and directional composition of MDA Top 100 versus DEG-only Top 100, GO Biological Process and Hallmark functional comparisons, and decomposed MDA/DEG-only projections. **Supplementary Tables S1**–**S13** cover data sources and participant information (S1), method parameters (S2), MDA Top 100 reference genes (S3), main GSEA results and direction scores (S4), TopN sensitivity (S5), gene-level similarity (S6), MDA/DEG-only comparisons (S7), random gene-set controls (S8), source data for **Figures 8**–**10** (S9-S11), terminology (S12), and the GSE164922 count-aggregation directional sensitivity analysis (S13). These materials support robustness assessment and clarify interpretive boundaries.
