# Supplementary material for: Reference-signature projection framework for exercise transcriptomic directionality based on the IAV-DI reference axis
Source: Front Physiol. 2026 Oct 7;17:1943015. doi: 10.3389/fphys.2026.1943015 (PMC13683089; doi:10.3389/fphys.2026.1943015)
Supplement: Supplementary file 1 [file SupplementaryFile1.docx]

***Supplementary Material***

# 1 Supplementary Data

This supplementary material supports the main results. It focuses on reference-signature robustness, random-background comparisons, the relationship between MDA and DEG-only ranking, and gene-set functional enrichment. It provides evidence for robustness assessment, random controls, MDA/DEG-only ranking differences, and functional enrichment support.

# 2 Supplementary Figures and Tables

This file contains Supplementary Figures S1–S6 with their captions. Complete supplementary tables are provided as a separate editable spreadsheet workbook.

## 2.1 Supplementary Figures

### Supplementary Result S1. TopN sensitivity analysis

To test whether direction-score results depended on the use of the top 100 reference genes, we repeated the analysis with the top 50, top 100, and top 200 reference genes. CPX showed weak positive values across TopN settings. Marathon changed more across TopN settings. This suggests that the CPX direction signal was relatively stable across reference-gene sizes, whereas Marathon was more sensitive to TopN selection.

**Supplementary Figure S1.** Direction scores across different TopN reference gene sets. The figure compares CPX and Marathon direction scores using Top50, Top100, and Top200 reference gene sets. Positive values indicate closer proximity to the pharmacological reference direction. Negative values indicate the opposite direction or no movement along that reference direction.

### Supplementary Result S2. Gene-level similarity plots

Gene-level quadrant plots visualize paired effect sizes for each MDA Top100 reference gene in the pharmacological reference and external disease or exercise comparisons. Each point represents one MDA Top100 gene. The x-axis shows the effect size in the pharmacological reference comparison. The y-axis shows the corresponding effect size in the external disease or exercise comparison. Red indicates positive concordance. Blue indicates negative concordance. Gray indicates discordant direction. The figure is used only to support gene-level interpretation.

**Supplementary Figure S2.** MDA Top100 gene-level similarity quadrant plots. The figure shows paired effect sizes of the same MDA Top100 reference genes in the pharmacological reference comparison and external disease or exercise comparisons. It supports interpretation of single-gene direction consistency.

### Supplementary Result S3. Random gene-set negative-control analysis

Random gene-set negative controls were used to test whether the NES values of the true MDA Top100 reference signature could be generated by random gene sets of the same size. Gray density curves show the NES background distributions from random gene sets. Orange vertical lines show the observed NES values of the true MDA Top100 reference signature. If the orange line is near the edge of the random distribution, the true signature shows stronger directionality than the random background. This analysis is a methodological negative control.

**Supplementary Figure S3.** Random gene-set negative-control analysis. Gray density curves show NES background distributions from repeated GSEA using random gene sets of the same size. Orange vertical lines show the observed NES values of the true MDA Top100 reference signature. The figure evaluates whether the true enrichment pattern differs from random gene-set backgrounds.

### Supplementary Result S4. Overlap and directional composition of MDA Top100 and DEG-only gene sets

To compare MDA ranking with DEG-only ranking, we summarize two results. First, Venn diagrams compare the overlap between MDA Top100 and DEG-only Top100 within Drug-UP and Drug-DOWN reference genes. Second, a directional composition plot shows the numbers of Drug-UP and Drug-DOWN genes in shared, MDA-only, and DEG-only-specific groups. MDA ranking retained part of the DEG-only core signal. It also retained more Drug-UP genes that were not prominent in DEG-only ranking.

**Supplementary Figure S4A.** Overlap of Drug-UP and Drug-DOWN genes between MDA Top100 and DEG-only Top100.

**Supplementary Figure S4B.** Directional composition of shared, MDA-only, and DEG-only-specific gene groups. The figure shows gene-composition differences between MDA and DEG-only ranking. DEG-only-specific indicates genes that entered DEG-only Top100 but not MDA Top100.

### Supplementary Result S5. Gene-set functional enrichment comparison

Functional enrichment analysis was used to test whether MDA Top100 and DEG-only Top100 captured the same biological processes and functional modules. This analysis describes functional tendencies of gene sets generated by different ranking strategies.

**Supplementary Figure S5A.** Gene Ontology biological process enrichment analysis. Dot size indicates the number of genes contributing to each term. Color indicates statistical significance. The figure describes functional tendencies of different gene groups at the GO biological process level.

**Supplementary Figure S5B.** Hallmark enrichment comparison. The figure summarizes shared and method-specific functional modules across different gene groups. It supports interpretation of whether MDA and DEG-only rankings capture the same functional modules.

### Supplementary Result S6. GSEA projection of MDA/DEG-only membership-derived gene sets

To explain the information provided by MDA ranking compared with DEG-only ranking, MDA Top100 and DEG-only Top100 were decomposed into shared, MDA-only, and DEG-only gene sets. These member gene sets were then projected into external disease, CPX, and Marathon comparisons.

**Supplementary Figure S6.** GSEA projection of MDA/DEG-only membership-derived gene sets. The figure shows NES values of different member gene sets in external disease, CPX, and Marathon comparisons. Tile color indicates NES. Asterisks indicate nominal FDR levels. The analysis supports interpretation of direction differences between MDA and DEG-only ranking.

## 2.2 Supplementary Tables

Complete supplementary tables are provided as a separate editable spreadsheet workbook. The workbook includes data sources and sample information, method parameters and thresholds, MDA Top100 reference genes, main GSEA and direction scores, TopN sensitivity, gene-level similarity, MDA/DEG-only controls, random-control summaries, pathway direction patterns related to main Figures 8–10, representative pathways, pathway divergence data, and abbreviations and term definitions consistent with the article.

| **Supplementary table** | **Content** | **Corresponding worksheet** |
| --- | --- | --- |
| Supplementary Table S1 | Data sources and sample information. | S1_Data sources and sample information |
| Supplementary Table S2 | Method parameters and thresholds. | S2_Method parameters and thresholds |
| Supplementary Table S3 | MDA Top100 reference genes. | S3_MDA_Top100 reference genes |
| Supplementary Table S4 | Main GSEA and direction scores. | S4_Main GSEA and direction scores |
| Supplementary Table S5 | TopN sensitivity analysis. | S5_TopN sensitivity |
| Supplementary Table S6 | Gene-level similarity. | S6_Gene-level similarity |
| Supplementary Table S7 | MDA/DEG-only controls. | S7_MDA_DEGonly controls |
| Supplementary Table S8 | Random-control summary. | S8_Random-control summary |
| Supplementary Table S9 | Pathway direction patterns for main Figure 8. | S9_Figure 8 pathway direction patterns |
| Supplementary Table S10 | Representative pathways for main Figure 9. | S10_Figure 9 representative pathways |
| Supplementary Table S11 | Pathway divergence for main Figure 10. | S11_Figure 10 pathway divergence |
| Supplementary Table S12 | Abbreviations and terms. | S12_Abbreviations and terms |
